# A Collaboration-Oriented M2M Messaging Mechanism for the Collaborative Automation between Machines in Future Industrial Networks

**DOI:** 10.3390/s17112694

**Published:** 2017-11-22

**Authors:** Zhaozong Meng, Zhipeng Wu, John Gray

**Affiliations:** School of Electrical and Electronic Engineering, University of Manchester, Oxford Road, Manchester M13 9PL, UK; zhaozong.meng@outlook.com (Z.M.); john.gray-2@manchester.ac.uk (J.G.)

**Keywords:** machine-to-machine (M2M), industrial internet of things (IIoT), industrial network, collaborative automation, interoperability

## Abstract

Machine-to-machine (M2M) communication is a key enabling technology for industrial internet of things (IIoT)-empowered industrial networks, where machines communicate with one another for collaborative automation and intelligent optimisation. This new industrial computing paradigm features high-quality connectivity, ubiquitous messaging, and interoperable interactions between machines. However, manufacturing IIoT applications have specificities that distinguish them from many other internet of things (IoT) scenarios in machine communications. By highlighting the key requirements and the major technical gaps of M2M in industrial applications, this article describes a collaboration-oriented M2M (CoM2M) messaging mechanism focusing on flexible connectivity and discovery, ubiquitous messaging, and semantic interoperability that are well suited for the production line-scale interoperability of manufacturing applications. The designs toward machine collaboration and data interoperability at both the communication and semantic level are presented. Then, the application scenarios of the presented methods are illustrated with a proof-of-concept implementation in the PicknPack food packaging line. Eventually, the advantages and some potential issues are discussed based on the PicknPack practice.

## 1. Introduction

The industrial internet of things (IIoT) and smart manufacturing have become the most popular industrial technical paradigms and business concepts in recent years. With the continuous integration of emerging information and communication technologies (ICT), the manufacturing industry is envisaged to experience a revolution in its way of operating toward autonomous and smart manufacturing. A wide spectrum of smarter sensors, actuators, controllers, and robotics are connected and integrated to constitute ubiquitous systems, which can potentially maximise the efficiency of machines and the throughput of operations [1]. The envisioned manufacturing systems can potentially empower collaborative manufacturing, which promises greater production flexibility and product variability with minimised human interventions.

As a new computing paradigm, the internet of things (IoT) has found applications in many different fields, such as home automation, medical aid, elderly care, smart grid, traffic management, and many others [2]. The integration of the IoT paradigm in the automation industry has fostered IIoT, which promises an even greater prospective future. There are several newly coined terms to describe this futuristic industrial concept, such as Manufacturing 2.0, Industry 4.0, Industrial Internet, smart factory and IIoT. Although they are proposed in a different context of applications, they share the basic ideas of improving the operation mode of industrial processes by interactive collaborations and intelligent optimisations of machines with connectivity.

Evidently, digital manufacturing is a highly interdisciplinary field involving a wide range of applied sciences. Studies in these fields have attracted much effort in both academia and industry, especially machine-to-machine (M2M) connectivity and communication which are critical building blocks for machine collaboration and process optimisation [3]. High-quality connectivity and ubiquitous M2M communications will boost innovations in industrial applications. However, industrial practices suggest that the organisation, collaboration, and interoperability of connected machines are still tricky tasks.

M2M service platforms are being standardised to enable inter-communication between machines at different levels, and many standard development organisations (SDOs) are working toward M2M standards for industry, such as the Institute of Electrical and Electronics Engineers (IEEE), the European Telecommunications Standards Institute (ETSI), the Internet Engineering Task Force (IETF), and the 3^rd^ Generation Partnership Project (3GPP). Some technological solutions and standards have been invented for different application contexts, such as Wi-Fi, 3G, 4G, Long-Term Evolution (LTE) radio communication standards, and network protocols WirelessHART, ISA100.11a, Z-Wave, and KNX [4]. There are also SDOs working toward system frameworks called manufacturing reference architectures (MRAs), such as the ETSI M2M service architecture, the Smart Manufacturing Leadership Coalition (SMLC) smart manufacturing platform, the oneM2M harmonised reference architecture, and Microsoft Discrete Manufacturing Reference Architecture (DiRA) [5]. The focal issues of these MRAs are openness, interoperability, inter-module collaborations, and manufacturing process optimisation.

Although the crucial technical issues have been addressed, there exists an underlying gap between model-based service architectures and mechanical operations. Thus, the techniques are not yet ready to use in dealing with the heterogeneous machines in a flexible and interoperable way. Ubiquitous M2M messaging and machine understanding are recognised as the central issues to manufacturing management. This article highlights the major technical gaps of M2M communications in IIoT, and gives a reference architecture and a collaboration-oriented M2M (CoM2M) mechanism to enhance the interoperability between interconnected machines focusing on machine connectivity, messaging and understanding.

## 2. The Vision of Future Manufacturing Industry Networks

It is predicted that there are over 50 billion connected devices in the world by 2020 [6]. The potentiality of IoT applications in diverse fields promotes the study and application of a wide spectrum of relevant techniques. The flexible connectivity, ubiquitous messaging, and interactive collaboration are the leading trend of future industrial computing paradigm. It allows greater machine efficiency and intelligent optimisations based on analytical insights of collected data of the products and manufacturing operations.

### 2.1. M2M Communications for Manufacturing Industrial Networks

**Figure 1:**
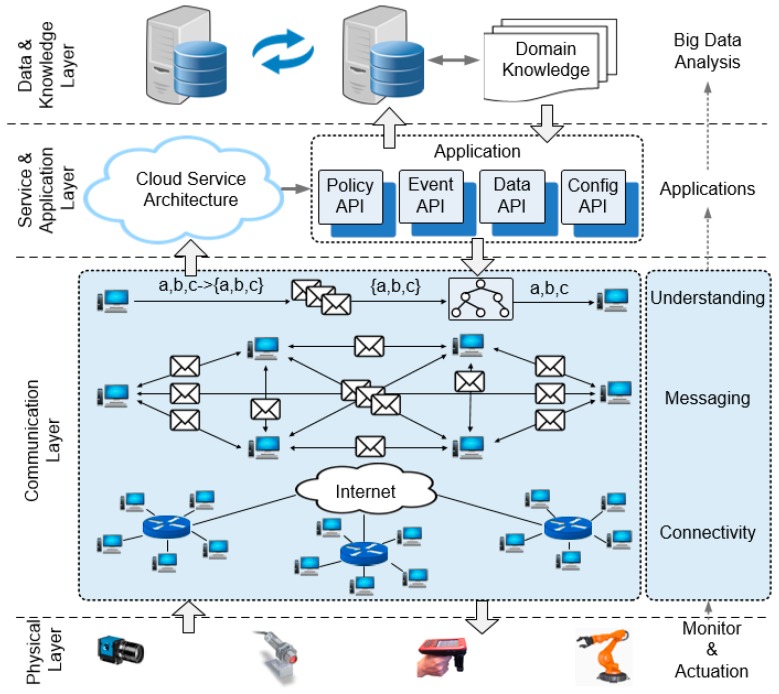
Machine-to-machine (M2M) communication in industrial internet of things (IIoT) systems.

Digital manufacturing integrates technologies of a variety of subjects, which are introduced to gain high-level automation and intelligence, such as radio communications, radio frequency identification (RFID), machine vision, interactive database, cloud service, and data analytics. To clarify the role of M2M communications in industrial applications, Figure 1 depicts the whole picture of an IIoT or digital manufacturing system. The underlying techniques are divided into four layers:

- The physical layer includes the functional machines that perform particular industrial operations, which are the basis to achieve autonomous operations. The smart sensors, actuators, robotics, RFID, machine vision, and precision control are the exemplar enabling technologies.
- The communication layer performs the machine communications for efficient data exchange and understanding. The radio communication technologies, and communication protocols and machine networking techniques are the building blocks.
- Service and application layer handles the events, processes the data, and deals with the logic of industrial tasks. Java Message Service (JSM), representational state transfer (REST), service-oriented architecture (SOA), and cloud service architecture are alternative solutions for the management of industrial operations.
- Data and knowledge layer conducts the analysis of produced data and discovery of rules to supervise the applications. ‘NoSQL’ solutions such as MongoDB and Rethink DB, and machine learning promise dynamic interaction and data analysis-based intelligence.

Since digital manufacturing is characterised by the automated configuration and autonomous collaboration of machines, the efficiency, reliability, and flexibility of machine connectivity and M2M communications become crucial parameters. There are two research focuses towards highly adaptive and responsive manufacturing. One is M2M protocols and connectivity solutions with network infrastructures based on Ethernet, Wi-Fi, cellular networks, and so forth. The other is software architecture and knowledge-based manufacturing management. Although M2M is widely accepted as a pivotal enabler of digital manufacturing, the M2M communications technology-based production line-scale machine interoperability has not been sufficiently addressed.

Evidently, the data interactions for the interactive functionalities including data collection, data request and response, events and commands notification, and execution of service layer functions are all through the intermediary communication technologies. Machine communications and understanding play an important role in the dynamic operations. There have been numerous investigations reported in the literature on how to apply existing standards and protocols that are suitable for industrial scenarios to achieve high-performance manufacturing management frameworks.

### 2.2. Enabling Techniques for M2M-Based Collaborative Automations

Due to the heterogeneity in machines and network connections, interoperability is a crucial parameter of IIoT systems. Allowing machines for cross-platform interoperable communications and data interactions is an important step toward machine collaboration in digital manufacturing. However, there are multi-fold meanings for M2M communications with respect to the hierarchical architecture of computerised systems, including connectivity and discovery, messaging mechanism, and semantic interoperability. The interoperability of the M2M communications is achieved through seamless integration of the underlying standards and protocols.

#### 2.2.1. Communication Level Techniques

With respect to communication level, many optional protocols, middleware, and application program interface (API) libraries are promising for M2M messaging. Although they are based on different techniques and for different application scenarios, the purpose is to gain flexibility in interactions between connected machines.

The main competitive candidates invented for different requirements and use scenarios are summarised in Table 1 [4,7]. Some are expert in handling lightweight messaging under varying levels of latency due to bandwidth constraints or unreliable connections, such as Message Queue Telemetry Transport (MQTT), and some are suitable for resource limited internet devices like wireless sensor networks (WSN) nodes in IoT applications, such as Constrained Application Protocol (CoAP). Others focus on data format, message orientation, queuing, routing, reliability, and security, such as Advanced Message Queuing Protocol (AMQP). There are highly capable techniques for supporting service discovery across network domains, such as Extensible Messaging and Presence Protocol (XMPP), which are well suited for cloud computing where virtual machines and networks would present obstacles. In addition, some are developed to simplify complex network programming for big data applications, like Data Distribution Service (DDS). There are also techniques providing easy connection, presence, and flexible development of application level functions like ZeroMQ (ZMQ). Since each solution has its strengths and particular suited fields, the selection of the alternative techniques should be based on the requirements of specific applications.

In the M2M messaging technologies, the information-centric networking (ICN) is an innovate networking paradigm in the IoT domain, which consists of the retrieval of content by names, regardless of origin server location, applications and distribution channels thus enables in-network caching and content-based security [8,9]. ICN is a new networking paradigm, which provides mechanisms that allow information to be exchanged using flexible semantics based information identifiers, rather than relying on end point, location dependent identifiers. Typical applications of ICN are information-centric IoT management architecture [10], ICN-based IoT architecture [11], and ICN-based M2M communications [12]. Although ICN is still in its early stage, it has attracted much research interest and is considered to be a promising networking technique to revolutionise IoT applications and reshape their future.

#### 2.2.2. Semantic Level Techniques

Semantic-level techniques are responsible for the understanding of messages and interoperability between machines, and therefore allow data to be shared and tasks to be collaboratively fulfilled across machines. This should be based on the normalisation of data and modelling of machine profiles, tasks, events, data interactions, and so on. For data normalisation, Extensible Markup Language (XML) and Javascript Object Notation (JSON) are widely used tools for the presentation of arbitrary data structures, which are already mature for data transfer of connected machines. This provides common frameworks to allow data to be shared and reused across applications with the standards to facilitate the data formats and exchange protocols.

For system modelling, ontology provides a formal naming and definition of types, properties, and interrelationships of the entities for a particular domain, which is widely used to categorise variables to establish the interrelationships for computations. The semantic data can be expressed through the definition of a common set of ontologies that describe the data items produced, exchanged, and consumed. For the IIoT, industrial scene, production policy, and machine configuration can be expressed with ontology concepts for computation and reasoning. An expressive ontology for IoT (IoT-O) has achieved device interoperability by extending one M2M standard [13]. An ontology-based smart production system for adaptive production management (ARUM) is discussed in [14]. Admittedly, ontology has been found a useful tool for specialised models or more general ones in IoT applications.

### 2.3. Open Research Issues

Future IIoT systems emphasise the autonomous nature of machines, and machine connectivity, messaging, and understanding are the central issues. Relevant investigations go in two focal directions: model-based service architecture for enterprise scale interoperability, and machine connectivity and communication networks and protocols.

Some model-based MRAs are proposed to pursue manufacturing flexibility, focusing on the modelling of goals, plans, capabilities, products, enterprise resources, business models, etc. Typically, the knowledge-based manufacturing reference architecture for collaborative production control [15], the Cloud Computing and Internet of Things–based Cloud Manufacturing (CCIoT-CMfg) service model integrating IoT and cloud computing for on-demand use and resources sharing [16], and ARUM system for adaptive and responsive manufacturing [14] are exemplar applications. In addition, a product lifecycle management (PLM) interoperability framework is proposed for collaborative product development by modelling of enterprise, business and information system [17]. A service-oriented architecture model is defined for coordinating execution of distributed automatic system management [18]. The relevant investigations focus on system level manufacturing management and address manufacturing flexibility at the enterprise scale with model-driven solutions, rather than the underlying machine flexibility in production line scale.

With respect to networks and protocols, SDOs and academic pursue interoperability mainly focusing on the communication and networking standards and architectures, such as IEEE 802.11ah for non-intensive M2M data exchange in industry [19], Bluewave wireless routing protocol for factory automation [20], M2M communication-based on 3GPP LTE/LTE-A networks [21], and CoAP/IPv6 over Low Power Wireless Personal Area Network (6LoWPAN)/REST for smart healthcare [22]. Since the application scenarios of manufacturing industry and some resource constrained device-based IoT applications differ in many aspects. The relevant studies do not achieve integral interoperability at the connectivity, communication, and semantic understanding as a whole to accommodate the specificities of industrial scenarios, and make it well suited for industrial applications.

In summary, there is a technical gap that restricts the data and information level technologies from truly integration with machine interactions and industrial operations to leverage machine interoperability and high level adaptability. To bridge the technical gap and achieve an interoperable manufacturing architecture, this investigation describes a collaboration-oriented M2M messaging mechanism toward machine interoperability for production line-scale machine collaborations in manufacturing industry and other IIoT applications.

## 3. Technical Solutions for Collaborative Automations between Machines

The collaborative automation between machines empowers highly adaptive and responsive industrial operations. Real-world industrial application features heterogeneity in machines types, operating systems and software platforms, and different application scenarios may differ in data throughput, interaction rate, real-time performance, and complexity of data structures, which makes many IoT solutions dedicated to lightweight devices and connections unsuitable. This section determines the specificity of industrial IoT applications and provides a reference system architecture with a proposed CoM2M messaging mechanism.

### 3.1. A Reference Architecture—Line, Modules, and Devices

**Figure 2:**
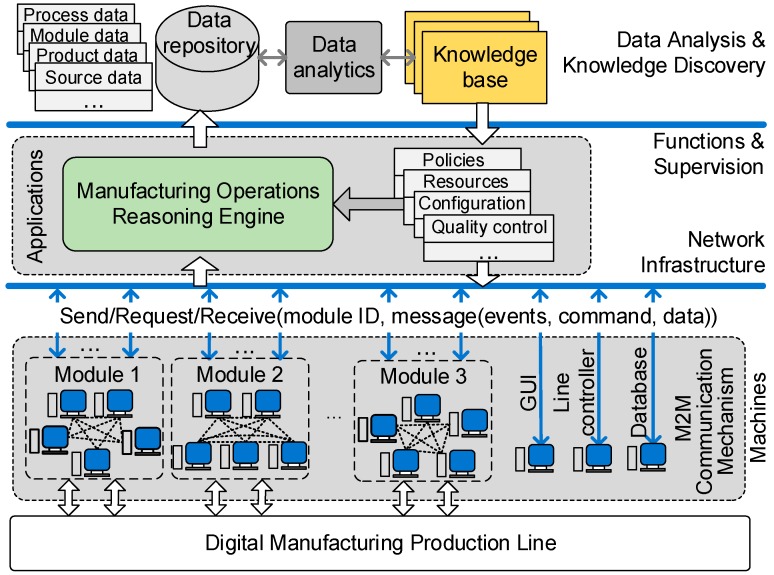
Reference System Architecture.

Different from self-organised ad hoc networks for WSN and lightweight computing devices, most digital manufacturing systems may find the following characteristics: powerful machines with reliable connection; heterogeneous machine platforms; ubiquitous messaging; high-throughput data transmission, and; data- and operation-centric tasks. Then, a reference system architecture is designed to depict the whole picture of manufacturing systems, as shown in Figure 2.

In the diagram, the machines are connected to a manufacturing network infrastructure to communicate with each other in an interactive way. The topology of machines in the network consists of three different hierarchies, namely, line, modules, and machines. The line includes all machines in the manufacturing line that fulfil all production processes and functionalities. The modules consist of one or several machines including a module controller to fulfil a comprehensive part of manufacturing functions. Then, devices are the basic functional units to achieve some specific functions.

The machines can talk to each other, and flexibly broadcast to one or more selected modules containing several machines. Evidently, the flexible messaging between machines allows the collaborations between machines to maximise their efficiency. The ubiquitous interactions also allow the collection and integration of product data. With effective notification of events and commands, machines can request data from peer machines and respond with data to requesters, and carry out the manufacturing operations in a collaborative and autonomous way. The flexible messaging also enables an interoperable information architecture which handles the real-time management of manufacturing operations by a reasoning engine with minimum human interventions.

Eventually, the collected data of products and operations can be used for data analysis, and the analytical insights can be used to optimise the processes and supervise the industrial operations.

### 3.2. Machine Connectivity and Messaging

#### 3.2.1. Machine Connectivity and Messaging Protocol

Due to the aforementioned features of digital manufacturing applications being non-lightweight, cross-platform, ubiquitous messaging, high-throughput, and so forth, Zyre—a ZMQ-based open source framework—is chosen to build the machine connectivity and implement the desired M2M messaging functions. The ZMQ Real-time Exchange Protocol (ZRE) governs how a group of peers on a network discover each other, organise into groups, and send each other events. ZRE runs over the ZMQ Message Transfer Protocol (ZMTP). Zyre is a framework for proximity computing over a local area network (LAN) like a manufacturing production line [23], which provides reliable group messaging over the industrial network with the following key features [24]:

- Needs no administration or configuration
- Machines may join and leave the network at anytime
- Machines talk directly to each other without central brokers
- Machines can join groups and then talk to groups
- Reliable even when the network is heavily loaded
- Fast and has no latency, requires no consensus protocols
- Designed for Wi-Fi networks, works on Ethernet networks.

The above features and the discovery and heartbeating functions make Zyre a competitive candidate for local service discovery and controlling a network of smart devices for IoT applications, including digital manufacturing. Since Zyre based on ZMQ is an application layer function library, it is convenient to develop applications and extend the functions on different platforms.

#### 3.2.2. Machine Discovery and Presence

**Figure 3:**
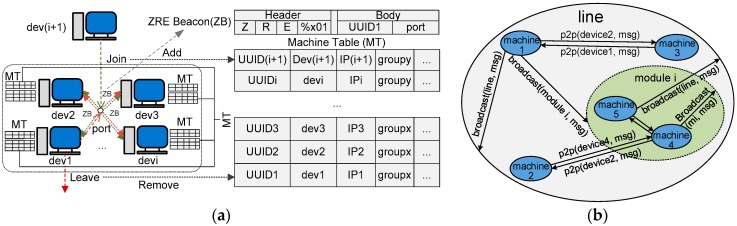
(**a**) Machine Discovery and Presence; (**b**) M2M Messaging Model.

With Zyre, machines can talk to one another and one-to-multiple grouped machines. Before communication, machines must be able to know all the connected peers available in the network and their states of connection. A unique device name and a universal unique identifier (UUID) for each machine are created to identify the machines in the network. The heartbeating mechanism allows the machines to notify their states to the peers and access the peers’ states as well. The fundamentals of machine discovery and presence can be described with Figure 3a.

As shown in the figure, Zyre uses IPv4 beacon broadcasts to discover machines. Each machine shall listen to the Zyre discovery service at a particular UDP port. Each machine shall broadcast, at regular intervals, on the UDP port a beacon that identifies itself to any listening machines in the network. In this case, each machine can maintain a lookup table containing information of all connected machines. The machines keep the local lookup table updated by being notified any events like machine joining in and leaving through beacon messages. In this way, machines make themselves known and discover information of all existing machines when joining in a network, and connected machines keep updated of all peers about their states.

#### 3.2.3. Ubiquitous M2M Messaging Functions

Enabled by the discovery and heartbeating techniques, machines can identify behaviours of peer machines such as entering the network, joining or leaving a group, and exiting the network. The machines can initiate or receive peer-to-peer (P2P) communications and broadcast through software functions ‘whisper’ and ‘shout’, respectively. As shown in Figure 3b, in the overall group ‘line’, there are machines and modules as sub-groups. The machines can talk to any single machine in the overall group using ‘whisper’. Machines can also broadcast to any group so that all machines in the group can receive the message. This versatile messaging mechanism makes data transfer and events notification flexible and convenient.

### 3.3. Message Events, Data Modelling and Presentation

In addition to flexible messaging, the definition of interrelationships between the connected machines and modelling of the events and functionalities are significant to the interoperable operations. Although the functionalities are application specific, appropriate modelling of communication level events and data presentation could promote the interoperability and machine understanding.

#### 3.3.1. Message Events

The machines, either devices or module controllers work as Zyre nodes. They send/receive events to/from the peer machines to handle the connectivity and data exchange. The control events for connectivity handling are ENTER, EXIT, JOIN, and LEAVE, which means entering an industrial network, exiting an industrial network, joining in a module group, and leaving a module group. The data events are WHISPER and SHOUT, which denote a P2P message and a one-to-multiple broadcast. The functions START and STOP are for the local nodes to start or stop a network connection. The manufacturing operations are based on ubiquitous messaging and the understanding of the message events.

#### 3.3.2. Data Modelling and Presentation

**Figure 4:**
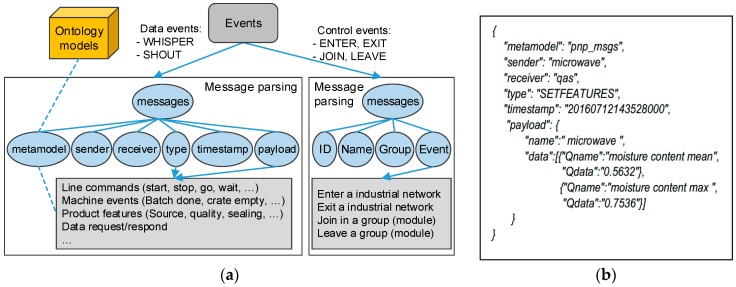
(**a**) Message Events and Data Modelling; (**b**) An Example of Javascript Object Notation (JSON) Description of a Message.

For interoperable understanding between the machines, the messages are normalised according to the model described in Figure 4a. According to the type of events, messages are divided into control events and data events. The control events are for network maintenance, which give the UUID, name, and group of machines and the type of control events. Data events enclose the descriptive metadata, such as where the message is from, where it goes, the type of message, the time stamp, and the ‘payload’ that includes the information structured according to its type. The receiver can parse the ‘payload’ node with the ‘metamodel’ and message type to obtain the useful data.

In order to accommodate the software environments of the heterogeneous machines, JSON is chosen for data presentation in machine communications. An example of a JSON-described message is as shown in Figure 4b. The tree-hierarchy data structure can be used to describe the messages. Since JSON is cross-platform for data presentation over the internet, the events, commands, and data can be structured and wrapped for the interactions between machines.

The proposed reference architecture is well suited for manufacturing applications to gain production line-scale machine interoperability. The decentralised structure of machine connectivity, autonomous machine discovery, ubiquitous messaging, and cross-platform presentation eventually contribute to the interoperability of manufacturing operations. The presented solutions are suitable to be used in dealing with the heterogeneity between machines, and therefore, data and information level methods can be introduced into industrial applications with these intermedium techniques.

## 4. PicknPack Practice

The reference architecture and CoM2M mechanism are a promising solution to deal with the interoperable M2M messaging and machine understanding in production line scale. The technologies can jointly contribute to the collaborative automations, and eventually allow industrial applications to be highly adaptive and responsive. The concept and the proposed methods are practiced and proved with PicknPack food packaging line as a case study.

### 4.1. Case Study: PicknPack Line

**Figure 5:**
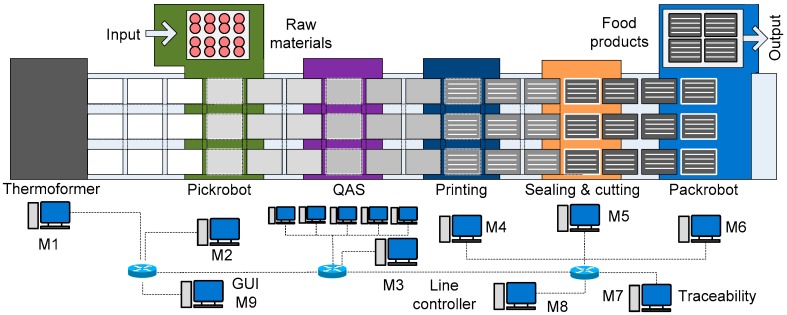
The PicknPack Food Packaging Line.

The PicknPack project aims to integrate the state-of-the-art robotics, sensors, and controllers with emerging ICT technologies to build a digital food manufacturing production line. The manufacturing operations include package making (Thermoformer), source food filling (Pickrobot), quality assessment and sensing (QAS), label printing, package sealing and cutting, finished product packaging (Packrobot), and information traceability. As shown in Figure 5, the workflow is as follows: the Thermoformer (M1) produces food packages; the Pickrobot (M2) picks up raw food materials and puts into the packages; the QAS (M3) gathers the quality information of food with five sensors (RGB, 3D, Hyperspectral, Microwave, X-Ray); the printing module (M4) then prints the labels; the sealing and cutting module (M5) seals the packages with printed labels and cuts them into individual food products, and finally; the Packrobot (M6) picks up the products and puts them into output crates for packaging and shipping. All the data of the products and industrial operations are collected and saved into the database by the RFID Traceability module (M7) [25]. The machines also listen to the line controller (M8) as a coordinator for collaborative operations and emergency handling. The machines in the manufacturing line perform the production operations in a collaborative way by the ubiquitous M2M messaging. For example, all other machines receive the encoder position messages from the Thermoformer to calculate the IDs of food packages in their workspaces and then perform their industrial operations and integrate the generated data. The Pickrobot fills the empty packages with raw food materials and integrates the raw material information with the package IDs. The QAS measures and evaluates the quality of food materials and integrates the quality data with their package IDs. The Printing machine requests raw material information, quality information, and other required information from Pickrobot, QAS, and so forth, with the ID of the package which is in its workspace and prints the information on the labels of the package. The Packrobot can request the quality information from QAS and classify the products into different outgoing boxes of different quality grades.

The manufacturing operations of PicknPack are implemented with the presented reference system architecture and CoM2M messaging mechanism. The Thermoformer notifies all the peer modules the encoder position using ‘shout’ for them to identify the IDs of packages in their workspaces. The modules can send messages using ‘whisper’ to request interested information with dynamically composed M2M messages for decision-making. All the machines save data locally and send to the traceability database for further use.

### 4.2. Demonstration and Evaluation

The PicknPack production line was successfully demonstrated in Wageningen UR, Netherlands, and National Centre for Food Manufacturing, Holbeach, UK in 2016. It is a successful practice of IIoT manufacturing concepts integrating a wide spectrum of technologies by gethering the industry and academic institutes.

In summary, the flexible M2M communications of PicknPack packaging line empowers the interactive collaborative operations between machines with the following advantages:

- Easy connectivity, machine discovery and presence
- Convenient machine network run-time maintenance
- Ubiquitous messaging for events notification and data exchange
- Flexible system configuration and reconfiguration
- Cross-platform interoperability between machines
- Real-time machine interactions for collaborative operations
- Open to support high level interoperable functionalities.

This M2M messaging mechanism is suitable for the collaborations of stationary industrial machines in a decentralised structure. Compared to those for lightweight self-organised ad hoc network or centralised infrastructure-based network, the strengths of the developed CoM2M lie in presence and discovery, ubiquitous messaging and machine understanding, which is well suited for IIoT manufacturing scenarios.

With the CoM2M mechanism, machines can easily build a connection, and initialise themselves with configuration files. They can also convinently initiate an event notification, data request, or data response in a flexible way. The whole line includes over 10 machines with LAN connection and the line moves forward in a speed of 7–15 cm/second. The size of JSON messages are in KB to MB. The latency of message is controlled in milliseconds in the operation of the system. The instant messaging and interoperable understanding between the machines gives them enough time to work on their manufacturing tasks.

**Figure 6:**
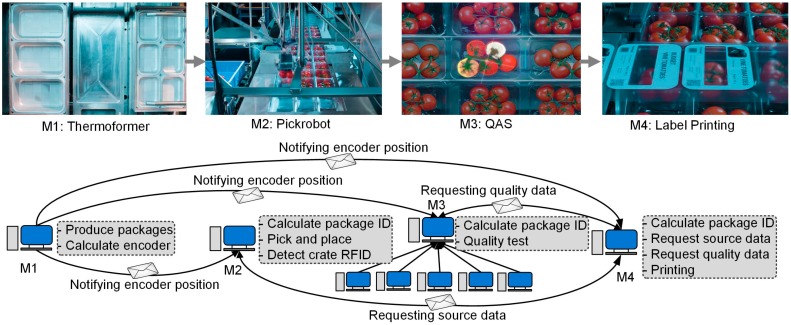
Machine Collaboration-based Dynamic Label Printing [26].

An example of M2M-based collaborative automation is the dynamic label printing of PicknPack food manufacturing, as shown in Figure 6. In the diagram, all modules (M1–M4) are notified the encoder position messages by the Thermoformer to identify the IDs of product packages in their workspaces, and perform their manufacturing tasks. The Pickrobot obtains the package IDs and link the information of raw food materials placed in the packages with the IDs. The QAS module obtains the package IDs and link the quality of food in the packages with the package IDs. When the Printing module identifies the IDs of product units in its workspace, it requests the source information from Pickrobot and quality information from QAS with the IDs, and then prints the information on product labels in real time. Therefore, the label printing operation is an interactive collaboration of four functional modules—Thermoforer, Pickrobot, QAS, and Printing with the underlying M2M messaging mechanism.

**Figure 7:**
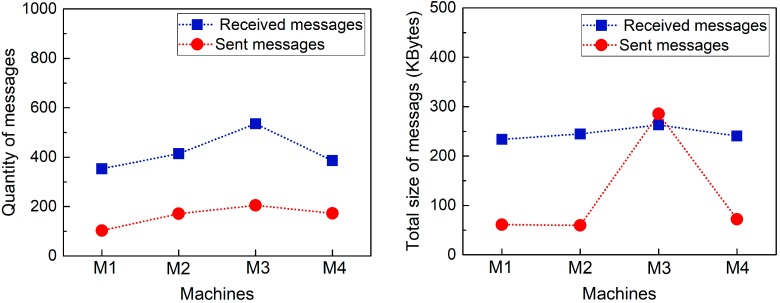
Quantity and Total Size of Messages Received and Sent by Machines (M1-M4) for Dynamic Label Printing.

Figure 7 gives the quantity and total size of messages received and sent by the machines performing the dynamic label printing task (M1-M4) in 1 min. The speed of messaging between machines is decided by the speed of manufacturing operations of the system. As shown in Figure 7, the QAS module receives and sends more messages than the peers since it coornidates five sensor machines and integrates their data to distribute to the machines of interest. For each machine, the number of received messags are much more than it sent because of the heartbeat messages from the peer machines. All machines receive heartbeat messages from the peers in the industrial network.

### 4.3. Potential Applications

Although the protocols and techniques for M2M communications may have overlapped application scenarios, they each have their specific fitting fields, such as ad hoc wireless sensor network, lightweight devices networking, P2P messaging over the internet, continuous data streaming transfer, and real-time collaborative operations. The industrial applications are characterised by high-performance computers with heterogeneous software platforms, which require real-time collaborative operations. Therefore, the described methods are designed for the practical manufacturing applications, which have the following features:

- Powerful machines with reliable connection
- High reliability in connectivity and messaging
- Ubiquitous messaging, either P2P or one-to-multiple
- High-throughput transmission and concurrent messages
- Awareness of the states of peer machines
- Real-time messaging for machine collaborations
- Highly dynamic and adaptive functionalities
- Decentralised machine network structure.

According to its features, the proposed CoM2M is an appropriate candidate for communication intensive smart machine integrated manufacturing applications, where sensors and actuators are applied to collect product data and perform automated production. In addition to the case study of PickNPack food manufacturing, it can find applications typically in mechanical manufacturing, pharmaceutical production, daily chemical goods production, aviation industry, and so forth, where machine parts for manufacturing are computerised and interactive machine collaborations are essential to the system.

## 5. Discussion

The practice of the presented methods in PicknPack line achieves production line-scale ubiquitous interaction and interoperable understanding. The performance of the system based on the presented technical solutions is encouraging. The lessons learned are summarised as follows.

A significant technical challenge is the integration of multiple levels applied technologies. This CoM2M messaging mechanism introduces platform-specific designs to comply with the Zyre-based messaging protocol in order to gain higher level interoperability. For end machines, APIs for the mainstream platforms C++, C#, and LabVIEW are developed for Ubuntu and Windows 7 as the major operating systems. If machines do not support third-party development, extra controllers need to be introduced as the intermediaries. Once all machines are compliant with the Zyre-based messaging protocol, machines can identify all peer machines in the network and initiate P2P or one-to-multiple messaging for data and events interactions. Some configurations, notifications, and requests can be accessed by all concerned machines with this messaging mechanism. In addition, the machines of PicknPack line are within a LAN, where ubiquitous interaction between machines is the major concern. With internet connection, manufacturing configuration and data maintenance can be deployed in a remote cloud service. For inter-enterprise interactive manufacturing which exceeds the scope of a LAN, further intermediary protocol adaptors need to be adopted.

Another crucial issue is the lack of common modelling formalism for the interoperable understanding between machines. The system supports flexible machine interactions, and therefore potentially promises flexibly manufacturing. However, the machine understanding, machine collaborations and the production flexibility need to be described with appropriate modelling, such as manufacturing policies and machine configurations. The PicknPack defines machine profiles, message formats, and machine configurations, and so forth, with a global description model named ‘world model’. All machines access the ‘world model’ for communal understanding to gain interoperability.

A further crucial issue in terms of flexible manufacturing based on CoM2M is the appropriate use of domain knowledge. Based on the interoperable technologies, some manufacturing functions fulfilled are application specific. Therefore, application specific knowledge such as the size and shape of packages, robot grippers, sensors, and quality evaluation methods for different food categories are all significant to the success of highly adaptive manufacturing. Such application-specific customizations are challenges for system developers.

For some IoT application scenarios, such as smart meter, smart home, smart healthcare, internet of vehicles, centralised system structure for lightweight devices and network connections might be of interest. The M2M solutions for these application scenarios may use publish/subscribe mode, and focus on their lightweight, mobility, and interoperability characteristics. Typical applications are lightweight XMPP publish/subscribe scheme [27], CoAP/Observer-based WSN [28], MQTT protocol for mobility in IoT [29], and AMQP/MQTT-based Message Oriented Middleware (MOM) protocols [30]. However, machines in manufacturing industrial networks require ubiquity of M2M messaging and machines interact with each other in an intertwined mode. It distinguishes with the others mainly in two aspects: (1) machines communicate with one another in an interactive and highly frequent way; (2) machines need data from peer machines to perform manufacturing operations. Therefore, it is a pivotal task to discovery and identify the machines, and talk to any machine in the network efficiently. Compared to some solutions based on the techniques listed in Table 1, the presented design of CoM2M featuring flexible discovery and presence, ubiquitous messaging, and semantic understanding is well suited for production line-scale intensive data interaction between machines in manufacturing industrial applications. The ICN networking paradigm is a promising technology for future large IoT systems in data distribution and security, which does not concentrate on the performance of a group of machines in a local area industrial network. The presented solution focuses on the interactive messaging and understanding for efficient manufacturing automations, which is a specialised design for production line-scale flexibility and interoperability.

## 6. Conclusions and Future Perspectives

The continuous technical progress has made it an overwhelming trend to integrate smart devices with emerging ICT techniques to reshape the industrial applications. M2M-enabled collaborative automation is a central technical issue to maximise machine efficiency and production flexibility. Motivated by the challenges in integrating data and information level techniques with low-level mechanical machines, this article discusses technical solutions toward machine interoperability in production line scale. Considering the specificities of industrial machines and the requirements, a reference system architecture and the CoM2M messaging mechanism are presented focusing on the connectivity, messaging, and understanding between machines. Its application in the PicknPack food packaging system has demonstrated the feasibility and its flexibility. The practice of the PicknPack system is a valuable reference solution for practitioners to innovate different industrial applications.

Along with the introduction of new technologies and concepts in manufacturing industry, such as IoT, wireless sensor networks, service-oriented technology, and big data, a lot of advanced manufacturing modes or national strategies have been put forward, including Industry 4.0, Flexible Manufacturing System (FMS), smart products, Made in China 2025, Internet Plus Manufacturing, and Cloud Manufacturing. Two technical issues will become the focal points of these new concepts of manufacturing: (1) the manufacturing efficiency based on data interaction and machine collaborations, and; (2) intelligent product and production process optimisation based on manufacturing data. The CoM2M presented in this paper allows real-time data interaction and collaborative automation between machines in order to promote the manufacturing efficiency of industrial networks with the solutions for machine network system modelling, flexible presence and discovery, ubiquitous M2M messaging, and machine understanding. The open framework also allows the system to implement high-level intelligence obtained from big data for production efficiency and product variability. With the progress of the computerisation of machine parts in the manufacturing industry, industrial networks will become more complicated, which may require the building of a connection with product designers, source providers, sales, markets, and customers, M2M messaging will become a challenge but also a driving force of future industrial IoT applications.

## Figures and Tables

**Table 1:** M2M messaging protocols, middleware, and application program interfaces (APIs). Standard Development Organisations (SDOs); Message Queue Telemetry Transport (MQTT); International Organisation for Standardisation (ISO); Organisation for the Advancement of Structured Information Standards (OASIS); Wireless Sensor Networks (WSN); Constrained Application Protocol (CoAP); Advanced Message Queuing Protocol (AMQP); Extensible Messaging and Presence Protocol (XMPP); Data Distribution Service (DDS); ZeroMQ (ZMQ); Internet Engineering Task Force (IETF); Object Management Group (OMG).

M2M Technique	SDO	Protocols/Middleware/APIs	Base Protocol	Licensing	Key Features
MQTT	ISO, OASIS	M2M connectivity protocol	TCP/IP	Formerly royalty-free license	Simple and lightweight For constrained devices and unreliable networks Message broker required
CoAP	IETF CoRE	Application layer protocol	UDP	Private/Liberal open-source license	Multicast, low overhead, simplicity, REST model-based For constrained nodes/networks
AMQP	OASIS	Application layer protocol	TCP	AMQP license	Message-oriented, queuing, routing, reliability, and security
XMPP	IETF, XMPP working group	Message-oriented middleware	TCP	GNU General Public License/proprietary	XML-based, decentralization, instant messaging, presence and collaboration
DDS	OMG	M2M middleware	TCP, UDP, HTTP	Open source/proprietary	Scalable, real-time, dependable, high-performance, and interoperable
ZMQ	iMatix	Asynchronous messaging library	TCP, UDP, PGM, IPC, ITC	GNU LGPLv3	Message queue without dedicated brokers, sockets supporting a many-to-many connection
